# *Implementation Science* and *Implementation Science Communications*: a refreshed description of the journals’ scope and expectations

**DOI:** 10.1186/s13012-021-01175-3

**Published:** 2021-12-04

**Authors:** Michel Wensing, Anne Sales, Paul Wilson, Rebecca Armstrong, Roman Kislov, Nicole M. Rankin, Rohit Ramaswamy, Dong ( Roman) Xu

**Affiliations:** 1 Heidelberg University Hospital, Heidelberg, Germany; 2 Sinclair School of Nursing and Department of Family and Community Medicine, University of Missouri, Columbia, MO USA; 3 Center for Clinical Management Research, VA Ann Arbor Healthcare System, Ann Arbor, MI USA; 4 Centre for Primary Care and Health Services Research, University of Manchester, Manchester, UK; 5 NIHR Applied Research Collaboration Greater Manchester, Manchester, UK; 6 Melbourne School of Population and Global Health, University of Melbourne, Melbourne, Australia; 7 Faculty of Business and Law, Manchester Metropolitan University, Manchester, UK; 8 School of Health Sciences, The University of Manchester, Manchester, UK; 9 School of Public Health, The University of Sydney, Sydney, Australia; 10 Cincinnati Children’s Medical Center Hospital, Cincinnati, USA; 11 SMU Institute for Global Health (SIGHT), School of Health Management and Dermatology Hospital, Southern Medical University (SMU), Guangzhou, China

## Abstract

This editorial provides a comprehensive consolidated overview of the scope and expectations of *Implementation Science* and *Implementation Science Communications*. We remain most interested in rigorous empirical studies of the implementation of evidence-based healthcare practices (including interventions, technologies, and policies) and the de-implementation of practices that are demonstrated to be of low or no benefit. Implementation strategies (e.g., continuing professional education, organizational changes, and financial incentives to enhance the uptake of evidence-based practices) are of central interest to the journals. We see the field as large and complex, with a wide literature that is published in many venues. We urge people for whom it is new to spend some time reading the existing literature, and learning the scope of the work that has already been done, and published, in our journals and in an increasing number of other journals in the field.

## Background

In 2006, the founding editors of *Implementation Science* declared that it was the journal’s mission to publish scientific contributions to implementation science in health, which they defined as “the scientific study of methods to promote the systematic uptake of research findings and other evidence-based practices into routine practice, and, hence, to improve the quality and effectiveness of health services” [1]. *Implementation Science* receives over 800 submissions annually and publishes around 120 papers. The number of downloads was 2.3 million last year. These numbers demonstrate the continued high interest among researchers, practitioners, managers, policymakers, and research funders.

Despite being an open-access journal, we inevitably have limited editorial and reviewer resources. A substantial proportion of the rejected manuscripts are in scope, scientifically sound, but do not meet the expectations of *Implementation Science*. We felt that these studies deserve to be published in an implementation science journal. In 2020, we launched the companion journal *Implementation Science Communications* to serve the same mission, but to enable consideration of a broader range of submissions [2]. *Implementation Science Communications* published more than 100 papers in its first year, more than we had anticipated. It is on target to receive about 400 submissions in 2021. The success of the journals has reinforced the need to reflect on the journals’ scope and expectations, a desire that was further enhanced by developments in healthcare and society—not least the global COVID-19 pandemic [3].

## Scope of the journals

This editorial does not represent a change in scope compared to our latest comprehensive editorial [4]. We remain most interested in rigorous empirical studies of the implementation of evidence-based healthcare practices (including interventions, technologies, and policies) and the de-implementation of practices that are demonstrated to be of low or no benefit*. Implementation strategies*, or implementation interventions, are of central interest: interventions that aim to enhance the uptake, sustainment, and scale-up of practices and those that aim to remove, reduce, replace, or restrict the use of ineffective interventions or practices. Our focus on health is broad and includes physical and mental health conditions, including addiction and other behavioral health areas. We consider clinical practice, preventive care, and health promotion interventions delivered in healthcare and other settings (e.g., schools and churches). The effectiveness of these interventions has been the topic of public health research, while other interventions have been examined in clinical or health services research.

We are open to a variety of methodological approaches, including qualitative, quantitative, and mixed-methods approaches. *Implementation Science* is particularly interested in studies that substantially advance the field by providing innovative, analytical, and generalizable insights (including non-mainstream approaches). *Implementation Science Communications* is interested in methodologically sound studies that contribute to new knowledge, which extend existing concepts and methods. Contributions may have a narrow focus in terms of clinical topic, patient population, or setting. In addition to empirical research, both journals publish systematic reviews and other types of contributions that we describe in the next section.

Our focus on implementation strategies means that we focus on approaches to enhance the uptake of evidence-based practices (e.g., continuing professional education, organizational changes, or financial incentives). Given the absence of widely accepted definitions of implementation strategies, we are open to various terms, including quality improvement, knowledge translation, and knowledge transfer. For instance, quality improvement is in scope, if the focus is clearly on the implementation of evidence-based practices. However, we do not publish on the implementation of research procedures (e.g., a randomization method). We recognize that implementation strategies are frequently applied in combination with clinical interventions, but we only consider studies that focus on the effects, processes, costs, and contextual factors associated with the implementation of these interventions. We focus on implementation strategies targeted at “agents of implementation,” such as health workers, patient navigators (usually volunteers), school teachers, and policymakers. This excludes most interventions to inform patients or populations about healthcare improvements or interventions to involve patients more actively in their healthcare (e.g., shared decision-making, patient adherence interventions, patient access to medical records). Such interventions are covered by other fields (e.g., clinical science, public health, or health services research). The implementation of these interventions may be in scope, if the evidence for the interventions is sufficiently robust, and the primary research question is associated with strategies to incorporate these into everyday clinical practice. The implementation of broadly phrased recommendations on desirable states in healthcare (e.g., “patient-centered care” or “holistic healthcare”) is out of scope, if specific strategies to achieve these states are not specified.

Outcomes of most interesting to us are observable aspects of healthcare delivery and healthcare providers’ behaviors, such as adoption, fidelity, penetration, and sustainability of changes in practice. These aspects may actually impact the health of patients and populations. We are less interested in studies that consider perceptions or cognitions of targeted individuals (e.g., perceived acceptability of interventions) as primary outcomes, but we do consider these in the context of process evaluations. We also are less inclined to publish studies that focus exclusively or primarily on clinical outcomes or patient-reported health.

The focus on *evidence-based practices* implies that we do not consider effectiveness trials of clinical and public health interventions, or other studies of interventions with uncertain effectiveness. Interventions for which evidence of effectiveness comes from a single study are borderline in scope. We prefer interventions that are supported by consolidated and synthesized research evidence, such as a systematic review or a systematically developed clinical guideline. We realize that emerging data-driven, individualized medical treatments (“personalized medicine”) may complicate the distinction between the generation and implementation of research evidence, and we will consider submissions in this domain on a case-by-case basis. We note that the use of an established framework that derives from implementation research (for instance, the use of CFIR in a process evaluation that is part of a clinical trial) does not equate to the study of implementation. Frameworks are increasingly used, particularly in the evaluation of novel interventions, but such use does not necessarily imply a contribution to the knowledge in implementation science (somewhat similar to the use of statistical methods in clinical research, which usually does not advance the methods themselves).

Better implementation of evidence-based practices frequently contributes to a reduction of disparities in health and greater equity in societies, given the unequal distribution of access to healthcare across subgroups in the population, as well as across nations. Health equity has become high on the societal agenda in recent years, which has also reinforced the attention for the topic in implementation science [5]. A substantial part of implementation science (implicitly) focuses on the reduction of health disparities through increasing access to recommended practices, but this needs to be done more systematically. We encourage authors to consider health equity in their submissions, and we may develop specific requirements in the future.

Given the rise of submissions on the implementation of digital technologies in health settings (e.g., video-based consultations, health monitoring through smartphone applications), we will elaborate on the journal scope in this domain. We will examine submissions in this field on a case-by-case basis. Given our focus on evidence-based practice, we would expect to see any technology underpinned by recognized evidence standards for effectiveness regarding the improvements on health and population outcomes (for instance, https://www.nice.org.uk/corporate/ecd7). We are interested in the implementation of evidence-based digital interventions, but not in the technical implementation of infrastructures (e.g., an online platform for delivering messages to patients’ smartphones).

Our interest in implementation interventions covers methods for the synthesis and dissemination of research evidence (e.g., the development of clinical guidelines), if the focus in the manuscript is clearly on consequences for implementation into practice. Mere analyses of change in healthcare practice (e.g., observed diffusion of innovations) are not considered. We also do not publish studies that merely document gaps between recommended and actual healthcare (“evidence-practice gaps”), but we do consider studies of factors associated with these gaps (e.g., “barriers and facilitators for implementation”) and studies that develop implementation interventions. Given the descriptive nature of many of the latter two categories (barrier identification and intervention development), many of these studies will be transferred to *Implementation Science Communications*. Given the increasing number of such studies, we will continue to reflect and possibly adapt our criteria for considering the publication of these studies.

Obviously, one of our priorities is to publish studies (and other types of articles) that have *added value* to the field of implementation science. Manuscripts with a strong analytical approach (e.g., new concepts or methods) and a study design that facilitates generalizability (e.g., multi-center cluster randomized trials) are likely to be reviewed at *Implementation Science*. Replication of previously conducted studies may also be considered, if the rationale is convincingly presented. Some manuscripts relate to implementation science, but they mainly have added value for a particular field of application (e.g., a specific healthcare setting). Others provide new insights or in-depth analyses, but have limited generalizability, given the study design, methods, or narrow focus. *Implementation Science Communications* welcomes such manuscripts, while *Implementation Science* is less likely to publish these. The boundary between relatively simple reports of lessons learned, with little to no analytic or theoretical insight, and small studies that provide thoughtful analyses, is not always easy to distinguish. At both journals, we receive large numbers of manuscripts in which little analytic insight is applied to the findings, and increasingly, these are being rejected at both journals.

## Boundaries of scope in relation to article type

This section provides further information on the boundaries of scope in relation to specific types of articles. The main considerations are summarized in Table 1. Except if indicated otherwise, the considerations apply to both journals.

**Table 1:** Summary of expectations of *Implementation Science* and *Implementation Science Communications*

	General expectations	Specific for ***Implementation Science***	Specific for ***Implementation Science Communications***
Theories and frameworks	Convincing rationale, based on comprehensive literature review	New, elaborated theories and frameworks of broad applicability	New concepts and theoretical perspectives and frameworks with broad or narrow applicability
Systematic reviews	Recent systematic literature search	Analytical depth and broad generalizability	Descriptive analysis or a narrower focus
Outcome evaluations	Focus on observable effects of implementation interventions	Randomized controlled trials and related quasi-experimental designs	Broader range of evaluation designs
Process evaluations	Related to implementation interventions of known effectiveness	Analytical depth and broad generalizability	Descriptive analysis or a narrower focus
Economic evaluations	Focus on the efficiency of implementation interventions	Cost-effectiveness and cost-benefit studies	Broader range of economic designs, including cost analyses
Implementation intervention development, pilot and feasibility studies	Focus on implementation interventions	–	Use of systematic methods and empirical research
Qualitative and mixed-methods studies	Chosen method matches with the focus of the study	Orientation on theory development, use of rigorous methods	Broader range of designs and methods to test theories
Quantitative observational studies	–	Study is guided by theory, use of advanced data analysis	Broader range of designs, including more pragmatic approaches
Validation studies	Comprehensive report of development and validation of measures	New measures	New, adapted, or translated measures
Study protocols	Funding from competitive funding programs, peer-reviewed	Multi-center randomized trials, and related designs	Broader range of study designs
Education on implementation science	Includes evaluation or other empirical research	Programs of substantial size and continuity	Broader range of educational programs
Methodology papers	Demonstration of method on study that is in scope of the journals	–	–
Commentaries and debates	Well-embedded in the implementation science literature	Potentially high impact on the field because of innovation or priority	Contribution with moderate impact or a narrower focus
Short reports	Comprehensive description despite shortness	–	–

### Theories and frameworks

Many theories and frameworks for implementation science are available. We are careful about adding new ones to the literature, as we feel that the field is better served by empirical testing of available theories and frameworks. Before we consider publication, the rationale for a new concept, theory, or framework (including extensions of published ones) needs to be convincingly presented. We welcome manuscripts adopting a critical approach to existing theories and frameworks, acknowledging their limitations (e.g., applicability in specific settings) and building on previous conceptual knowledge to develop new generalizable insights from empirical data. However, it is essential that a comprehensive (ideally systematic) review of existing theories and frameworks is included in any critical reflection or proposal for a new theory. When deploying existing theories and frameworks in studies, the authors should ensure that these are not applied in a superficial, tokenistic fashion. Instead, we recommend an in-depth engagement with selected theories and frameworks throughout the manuscript. Both journals are increasingly reluctant to publish studies that categorize data according to a framework without offering interpretations that relate to the underlying theory. Theories and frameworks should explicitly inform research aims and objectives, guide data collection and data analysis, shape the presentation of findings, and provide a basis for articulating the study’s contribution in the discussion section.

### Systematic reviews

We publish various types of reviews of published research, including traditional (“Cochrane style”) systematic reviews, scoping reviews, syntheses of qualitative research, and rapid reviews. At a minimum, the literature search needs to be transparent and generally exhaustive, cover at least three databases, and not be more than two years old at the date of submission.

### Outcome evaluations

Outcome evaluations are studies that aim to determine the effectiveness of, or changes associated with, implementation interventions. We consider hybrid designs that place emphasis on the evaluation of implementation outcomes rather than clinical or population health effects. Evaluations of novel or existing interventions with limited or no evidence are not considered. We welcome outcome evaluations that show that implementation strategies had little or no impacts, provided that the rationale for the chosen strategies was plausible. As outcomes, we prefer validated measures of observable aspects of healthcare delivery or professional performance, which have clear relevance for clinical and population health. Studies reporting only self-report outcomes are unlikely to be considered. Studies that exclusively examine the effects of interventions on participants’ knowledge, cognitions, or perceptions are unlikely to be considered as outcome evaluations, but we may consider these as process evaluations. For outcome evaluations, *Implementation Science* expects a study design that optimizes internal validity, such as a (cluster) randomized trial. We regard pragmatic trial designs as important in implementation research. The journal also considers rigorous non-randomized controlled studies, difference-in-differences, and interrupted time-series designs, if a clear rationale for the choice of study design is provided. *Implementation Science Communications* considers a broader range of designs for outcome evaluation, including uncontrolled before-after comparisons when well justified. However, weak designs and poorly conducted studies will likely be rejected by both journals.

### Process evaluations

Process evaluations of implementation interventions examine the degree of uptake of implementation interventions, processes, and factors associated with changes in implementation outcomes (or absence of it), side effects of the interventions, and/or factors that influence the sustainability and scalability of the implementation interventions. Both journals expect that the focus is on implementation interventions of known effectiveness, so that the findings of the process evaluation explain and contextualize the findings of the main study. We reject a large proportion of submissions because the effects of the implementation interventions of interest are not reported (elsewhere or in the same manuscript). Process evaluations of complex clinical or public health interventions, which have a strong implementation science component and are part of large multi-center cluster randomized trials, are considered on a case-by-case basis. *Implementation Science* prefers the use of a theory or framework for the guidance of the process evaluation and a study design that facilitates generalization. *Implementation Science Communications* also considers process evaluations that are more pragmatic or less generalizable. Within these parameters, both journals welcome submissions of process evaluations that help to explain null results of trials as well as those with positive findings.

### Economic evaluations

We welcome economic evaluations of implementation interventions, preferably those that analyze both effects and costs. Cost-benefit and cost-effectiveness evaluations of implementation interventions essentially need to meet the same requirements as outcome evaluations. Manuscripts that describe cost analyses (ignoring effects) are only considered if the effectiveness of the implementation interventions is reported (this is similar to process evaluations). Cost analyses without examinations of outcomes are usually transferred to *Implementation Science Communications*.

### Implementation intervention development and pilot and feasibility studies

*Implementation Science Communications* welcomes manuscripts on the development, pilot testing, and feasibility of implementation interventions. Few if any of these will be published in *Implementation Science*. To be considered, intervention development and testing should be based on systematic methods and, at least to some extent, empirical research in healthcare settings. They must be focused on implementation interventions, not other types of intervention (clinical, system, or other novel interventions). We prefer comprehensive reports of intervention development and testing over manuscripts that focus on specific aspects of the intervention development.

### Qualitative and mixed-methods studies

We welcome qualitative and mixed-methods studies but have rejected many because of poor methodological design, conduct, and reporting. Like in all research papers, the methods need to be specified and described in sufficient detail to be able to understand what has been done. We are open to a variety of qualitative methods, but it is important that the chosen method matches the research question and field of application. For instance, it may be hard to justify a purely inductive analysis in a study of barriers for implementation, given the large number of available conceptual frameworks that could be used to categorize the items. *Implementation Science* prefers studies that relate to existing theories, frameworks, or concepts, seeking to yield new theoretical insights applicable to a wide range of settings and useful for guiding future empirical enquiry [6]. *Implementation Science Communication* also considers more pragmatic studies, although lack of any theoretical insights is increasingly resulting in rejection, often with a recommendation to revise and provide stronger analytical and theoretical insights.

### Quantitative observational studies

We welcome theory-guided, quantitative observational studies, which use up-to-date or advanced analysis methods to explore or confirm determinants, processes, costs, and mechanisms of implementation. Quantitative observational studies may use concepts and methods from a variety of sciences, such as psychology, economics, and sociology. Some of these studies are (quantitative) process evaluations of implementation interventions. Quantitative observational studies that are outcome evaluations (e.g., uncontrolled before-after designs) are usually not considered by *Implementation Science*, but they may be considered by *Implementation Science Communications*. In all studies, we prefer that the study protocol and data analysis plan are pre-specified. We are increasingly inclined to reject studies that pick a framework and mechanically present measures or data categorized by its constructs, yielding little to no further analysis.

### Validation studies

An increasing number of submitted manuscript report on the development and validation of measures of implementation constructs. This is an important type of research, because valid measures are required for scientific progress. *Implementation Science* is particularly interested in the validation of new measures of implementation constructs, except if many similar measures have already been validated. *Implementation Science Communications* also considers manuscripts on the adaptation and validation of existing measures for specific healthcare settings, countries, or languages. In both journals, we prefer comprehensive reports rather than a series of incremental reports about parts of the validation study.

### Study protocols

We publish (without external peer review) protocols that have been through competitive peer review to receive funding from a nationally or internationally recognized research agency and that have received ethical approval or exemption. We will publish these increasingly in *Implementation Science Communications*, but *Implementation Science* remains interested in study protocols of large and innovative research projects or programs of research that are submitted within 12 months of ethical approval. We require prospective registration of all studies meeting the WHO definition of clinical trials (https://www.who.int/health-topics/clinical-trials#tab=tab_1), and note that we include behavior change, on the part of providers as well as patients, to constitute outcomes under the WHO definition. We continue to reflect on our policies regarding study protocols, with a particular view on countries that offer little opportunity to get funding from recognized research agencies.

### Education on implementation science

The field of implementation science needs well-trained researchers and practitioners. Therefore, we also consider manuscripts concerning education and building capacity in implementation science, provided that they include empirical research—ideally, longitudinal evaluation of a program [7]. We prioritize manuscripts on rigorous evaluations of education programs of substantial size and continuity over manuscripts on single courses or sessions. As the body of research literature concerning education on implementation science is growing, it is also important that the manuscript is well-embedded in this literature.

### Methodology papers

Both journals welcome contributions on research methods, which are relevant for implementation science (although they may not be unique to implementation science). We prefer that the use of these methods is demonstrated through concrete examples, which relate to implementation science rather than other fields and are generally in scope for both journals. Both journals usually reject descriptive accounts of largely established methods without any associated novel methodological insights.

### Commentaries

Commentaries are considered if they convey new or important ideas on implementation science. We carefully consider whether the contribution is sufficiently embedded in the implementation science literature. Some commentaries have been invited, because the journal editors feel that specific themes need to be highlighted. We also receive unsolicited commentaries, and have published a few, but we often feel that the added value is limited and inadequately contextualized in the existing literature. We therefore strongly recommend making an enquiry with the senior editors before making any submission. The journals will continue to invite commentaries on specific topics, which are internally prioritized.

### Debate

We receive numerous debate manuscripts at both journals. We welcome debate and discussion in the field and review these carefully. However, many submissions provide only highly selective reviews of the literature, often setting up arguments that are easy to critique. We commonly reject these. As the field is evolving, we consider it essential to ground arguments in a full view of an increasingly large and complex literature, so we strongly recommend that the authors conduct a careful and comprehensive (ideally systematic) review of the literature before drafting a debate manuscript.

### Letters to the editor

We encourage the use of letters to the editor, noting that they should be short (800 words or less), and focus on a critique of a specific published paper in the journal to which they are submitted. Letters that address issues from multiple manuscripts without a clear focus on a specific paper will likely not be accepted as letters. As with all publications, letters to the editors will be peer reviewed.

### Short reports

Manuscripts on empirical studies can also be submitted as short reports, provided that they are sufficiently comprehensive despite their format. Given its focus on sound research with moderate added value, *Implementation Science Communications* encourages the use of short reports as well as the use of online additional files that provide added information.

## Reporting expectations

Both journals have a number of expectations regarding the reporting of studies, which are summarized here. We expect that authors adhere to the principles of scientific integrity, including those that apply to the authorship of scientific papers. We perceive a trend towards increasing number of authors on manuscripts, but it is not feasible for journal editors to examine whether all fulfill the requirements for authorship. We therefore expect those designated as authors to demonstrate that they meet all four ICJME criteria for authorship (http://www.icmje.org/recommendations/browse/roles-and-responsibilities/defining-the-role-of-authors-and-contributors.html). For manuscripts with large numbers of authors (e.g., 20 or more), we increasingly ask that a writing group is named that then appears in the article byline. We appreciate that authors may be concerned that their contribution will not be recognized, but MEDLINE indexing can handle group authorship. We also recognize a growing trend towards the designation of more than one “first” or “last” author. We do not explicitly make these designations as part of our publications. Regarding submissions from low- and middle-income countries, we recommend to include at least one author from the countries of interest; these authors should obviously meet the ICJME criteria for authorship. We expect ethics approval (or a waiver/exemption) by a recognized, independent ethics committee for all empirical studies. Authors of all empirical studies and systematic reviews are requested to use an appropriate reporting guideline, most of which can be found online (www.equator-network.org). We require prospective registration in a recognized trial register for all studies that meet the criteria for clinical trials according to WHO or NIH (essentially all prospective studies of interventions in humans). For other studies, particularly studies that are designed to test hypotheses and systematic reviews of intervention studies, prospective registration is strongly encouraged and may become obligatory in the future.

Empirical studies should obviously be scientifically sound, which implies that objective, methods, results, and conclusions are adequate and logically linked. As journals, we remain committed to improving the quality of intervention description; implementation strategies are often inconsistently labeled and poorly described. Without sufficient detail, it can be difficult for readers to determine what was actually implemented or for researchers to use or replicate a strategy in other studies. TIDieR [8] is most often used for intervention reporting in trials [9]. Other frameworks which call for more detailed specification of both strategies and the behaviors to be targeted should be considered [10].

Implementation science is a fast-moving field, and we expect all manuscripts to be well-embedded in the contemporary literature. For empirical papers, this requirement relates both to the literature review presented in the front end of the manuscript and to the discussion section, which should aim to interpret the findings in light of relevant scientific literature rather than merely describe empirical results. We receive many submissions across both journals which do not fulfill this criterion and that are either sent back to authors or deemed to be of insufficient priority for review. Both journals are international in scope, which implies that authors are expected to relate their study to the global scientific literature or fully justify why orientation to a single jurisdiction is necessary. In addition, sufficient contextual information is essential for readers from other jurisdictions. We also encourage the authors to acknowledge the limitations of their studies, articulate their implications for policy and practice, and outline directions for future research. Finally, we require that each manuscript includes 2–3 statements on what this study adds to knowledge and understanding in implementation science. Authors will be increasingly requested to revise these statements, if they do not provide pertinent information. These should not be duplicative of the abstract.

For a regular research paper, we allow a maximum of 5500 words but encourage authors to use fewer words as we believe that many readers prefer concise papers. The number and size of supplements to a manuscript are unlimited, and these can be used to provide additional information.

## Reflections on the relationship between *Implementation Science* and *Implementation Science Communications*

Our primary purpose in launching *Implementation Science Communications* was to support publishing a broader variety of papers reporting on aspects of the science of implementation. We are still working out clear principles for transfer, many of which are articulated below. It is important to note that transfer from *Implementation Science* to *Implementation Science Communications* does not imply that a manuscript will be accepted for publication, although the likelihood of review is higher than average. We make independent editorial decisions at both journals and may deem a manuscript out of scope when assessed at *Implementation Science Communications* even when it is transferred from *Implementation Science.* In addition, although we make every effort to streamline processes for transferred manuscripts, if comments were provided before transfer, and particularly if the manuscript was reviewed, we expect that appropriate revisions will be made prior to transferring to *Implementation Science Communications*.

It is also worth reiterating that *Implementation Science Communications* is committed to sound science [2]*.* While the identity of *Implementation Science Communications* as a new journal is still emerging, our intention is to promote the development of its distinct scope, contributing to advancing the field in the following directions. First, in its openness to publish smaller-scale empirical contributions, pilot studies, and alternative viewpoints, the journal aims to increase the diversity within the field, helping new ideas enter the mainstream. Second, by inviting research conducted outside healthcare settings, *Implementation Science Communications* aspires to facilitate dialog among implementation scholars working in a wider range of settings, such as education, social services, and public policy, and thus better understand sector-specific contextual influences on implementation. Finally, *Implementation Science Communications* encourages submissions that engage with theory, either through theoretically informed or theoretically informative approaches [6], but do so in a practice-oriented way, whereby theory is not only deployed for its own sake but assists in understanding and solving “real-world” implementation issues.

### Final considerations

As we note, implementation research is a rapidly growing field, with growing efforts to dedicate funding in some jurisdictions. This draws new entrants into the field, many of whom come from other health research backgrounds, and for whom implementation science seems novel and highly emergent. With over 15 years of publishing *Implementation Science* and now also publishing *Implementation Science Communications*, we see the field as large and complex, with a wide literature that is published in many venues, and urge people for whom it is new to spend some time reading the existing literature, and learning the scope of the work that has already been done, and published, in our journals and in an increasing number of other journals in the field.

## Conclusion

This editorial described the mission, scope, and expectations of *Implementation Science* and *Implementation Science Communications* and highlighted some differences between the journals. We intend to support authors in their consideration on whether to submit to the journals, and hope we will continue to receive many high-quality submissions in the coming years.

## Data Availability

Not applicable.
